# Birth Defects in Singleton versus Multiple ART Births in Japan (2004–2008)

**DOI:** 10.1155/2011/285706

**Published:** 2011-11-24

**Authors:** Syuichi Ooki

**Affiliations:** Department of Health Science, Ishikawa Prefectural Nursing University, 1-1 Gakuendai, Kahoku, Ishikawa 929-1210, Japan

## Abstract

The purpose of the present study was to evaluate the relative risk (RR) of multiple births for birth defects after assisted reproductive technology (ART) using Japanese nationwide data from 2004 to 2008 with singletons as the reference group. In multiples compared to singletons, the percentage of birth defects per pregnancy were significantly higher (RR = 1.88, 95% confidence interval (CI) 1.60–2.13), the percentage of birth defects per live birth was not significantly higher (RR = 0.90, 95% CI 0.78–1.05 or RR = 0.94, 95% CI 0.81–1.10), and the early neonatal mortality rate was significantly higher (RR = 2.68, 95% CI 1.52–4.70 or RR = 2.80, 95% CI 1.60–4.92). The early neonatal mortality per 10,000 live births was slightly higher in ART (5.09) than in the general population (3.86). We concluded that the impact of birth defects after ART would be larger in families with multiples compared to families with singletons, since the mean number of children would be larger in the former.

## 1. Introduction

The possibility of an association between assisted reproductive technology (ART) and birth defects was raised as early as 1987 [1]. In more recent years, as the number of children born after ART increases, larger studies with more robust methodologies have suggested that children born after ART have an increased risk of birth defects compared with children conceived spontaneously [2, 3]. 

Data from recent meta-analyses consistently suggest that the overall risk of major birth defects in children born after ART is about 30% higher than in children conceived spontaneously [4, 5]. A more recent nationwide survey in Sweden also showed a slightly increased risk for birth defects after in vitro fertilization (IVF), even adjusting for possible confounding factors, such as year of birth, maternal age, and parity [6]. On the other hand, the first large-scale report of birth defects in 15,405 offspring conceived by ART in China found that infants born after IVF/ICSI have a birth defect frequency comparable to that in the general Chinese population [7]. 

Many studies and meta-analyses have shown an increased risk for singletons conceived by ART [8, 9]. It is controversial whether there is a risk to twins born after ART [9–11], in part because of the fact that ART usually produces dizygotic twins. It is well known that the medical outcome of dizygotic twins is better than that of monozygotic twins [12]. According to the meta-analysis by McDonald et al. [13], there were no significant differences in the number of birth defects between spontaneous and IVF twins. A recent systematic review comparing the data between spontaneous and ART twin pregnancies/neonates showed that the birth defect rate was equal in 7 of 9 studies [11].

It has been established that the prevalence of birth defects is higher in multiples than in singletons in total (not stratified by the method of conception), as shown in national studies [14, 15] and an international study [16]. As is well known, multiple births occur far more often in ART than spontaneous conception in almost all developed countries [17–24]. The multiple-birth rate (per 1,000 live births) increased twice during the past two decades, mainly due to the increase of iatrogenic multiples, including ART in Japan [24]. Therefore, the effect of multiple births should be considered when estimating the effect of ART on birth defects.

According to the Japanese ART and vital statistics, which cannot be directly linked, the percentage of ART live births were 1.64% (18,168/1,110,721) in 2004 and 1.99% (21,704/1,091,156) in 2008. Thus, the use of ART is becoming widespread in Japan, but there are very few epidemiologic reports on ART. One reason is because the collection of the data on ART and birth defects in Japan is not systematically managed by the government but by several academic societies and it is very difficult for researchers to access personal information. The largest database on ART is managed by the Japan Society of Obstetrics and Gynecology (JSOG), as mentioned in detail in Section 2. The largest birth defects data is managed by the International Clearinghouse for Births Defects Monitoring Systems (ICBDMS) Japan Center, which is a volunteer hospital-based registry covering about 10% of all births in Japan. However, this database does not have detailed information on ART. Vital statistics (birth records) from the Ministry of Health, Labour and Welfare of Japan also list the number of birth defects in neonatal/infant mortality but with no information on ART. These three possible data sources are managed independently, and record linkage is virtually impossible. With these essential limitations on the data collection, the present study was performed. 

Considering the increasing use of ART, the effect of multiple pregnancies in ART should be properly estimated. The purpose of the present study was to offer an overview of birth defects after ART in Japan and to evaluate the relative risk (RR) of multiple births, with singletons for reference, on birth defects using Japanese nation-wide data.

## 2. Methods

### 2.1. Outline of Japanese ART and Birth Defects Data

#### 2.1.1. ART Data

ART began in 1983 in Japan. Almost all medical institutions performing ART are registered with the JSOG. The JSOG administers questionnaire surveys for these medical institutions. Some of the survey data are presented in simple annual reports of aggregate, not individual, data (with no information available in English). The JSOG has gathered ART data from registered institutions since 1985. The data items were not necessarily constant throughout the surveillance period.

From 2004 to 2008 (the latest), the individual list of all ART pregnancies having birth defects was presented every year in the above-mentioned ART annual reports. The presented items are method of treatment (IVF, microinsemination (ICSI), frozen embryo transfer and others (duplicative methods), and do not include simple ovulation stimulation/enhancement), maternal age, perinatal outcome (spontaneous/artificial abortion (<22 weeks), stillbirths (≤22 weeks), and live births) and their gestational week, plurality (singleton, twins, triplets/+, and unknown), sex (male, female, unknown), early neonatal infant death up to day 6 (yes, no, unknown), and name of disease of birth defects, including chromosomal abnormality (with no clear definition or inclusion criteria for the birth defects). 

The author used these data as initial information for the present analyses. All methods of fertility treatment were treated as ART in the present study, because the classification of these methods is not necessarily consistent and mutually exclusive. 

The response rate for ART surveillance between 2004 and 2008 was 97.7–99.5%, and the mean response rate throughout the 5 years was 98.9%. In total 1,167 abortion, stillbirths or live births with birth defects, consisting of 934 (80.0%) singletons, 218 (18.7%) twins, 11 triplets (0.9%), and 4 (0.3%) unknown, were reported. Twins and triplets were treated in one category as multiples in the present study, since the total number of ART live/stillbirths according to subtype of multiples were not obtained.

#### 2.1.2. Estimation of the Numbers of ART Singletons and Multiples

The number of pregnancies, that is, the number of total women with gestational sac, after ART was presented according to plurality in the ART data. As for the ART births (including both stillbirths and live births) data, the total number of live deliveries and stillbirth deliveries, that is, the number of total mothers, and live-births, that is, the number of total neonates, were the only available data from 1989 to 2008. The numbers of multiple live-births according to subtype were presented only in the 2007 and 2008 surveys. For the multiple pregnancies, the mothers who had at least one live-birth neonate were counted as a live delivery and the others as stillbirth deliveries. For the multiple births, the births were counted as live only when all neonates were born alive. With these data limitations, the author estimated the minimum and maximum numbers of ART singletons and multiples between 2004 and 2006 using approximation formulae [25].

### 2.2. Statistic Analyses

First, demographic and perinatal outcome data of the subjects were calculated. For the comparison between singletons and multiples, the *t*-test was performed for maternal age and gestational weeks and the *χ* ^2^ test for birth year, sex, and perinatal outcome. The significance level was set at  .05.

Next, the crude percentage of birth defects per ART pregnancies and ART live-births were calculated according to plurality and their RR with the corresponding 95% confidence interval (CI). The crude percentage of ART early neonatal deaths with birth defects in live-births according to plurality and their RR with the corresponding 95% CI were also calculated. The crude percentage of birth defects per total ART births, including both stillbirths and live-births, were calculated, since the total ART births according to plurality were not obtained. The comparison between ART and the general population (vital statistics) concerning the crude early neonatal mortality (within 7 days after birth) rate related to birth defects (per 10,000 live-births) was performed.

Stepwise multiple logistic regression analyses were performed to determine which factors influence the neonatal outcome of birth defects (stillbirths versus live-births, and if live-births, whether early neonatal death occurred or not), with a threshold significance level of  .05. The variables used were birth year, plurality, maternal age, sex, and gestational week. All statistical analyses were performed using SAS for Windows ver. 9.2.

## 3. Results

Demographic and perinatal outcome data of ART pregnancies with birth defects are summarized according to plurality in Table 1. The percentage of multiple births in each year tended to decrease significantly from 2004 to 2008. Maternal age was significantly higher in singletons. Gestational weeks were significantly longer in singletons. There was no significant sex difference. Males were more frequent in both singletons and multiples. Live-births were more frequent in multiples, while early neonatal death was also more frequent in multiples. Unknown/missing values of early neonatal death in singletons were very high (26%). 

The crude percentage of birth defects in ART pregnancy according to plurality are shown in Table 2. The percentage increased with birth year. The percentages in multiples were consistently higher than in singletons. RR is around 2 and is statistically significant throughout all 5 years (RR = 1.88, 95% CI 1.60–2.13 in total).

The crude percentage of birth defects in ART live-born babies are shown in Table 3. The percentage and RR slightly and consistently increased with birth year. The percentages of multiples were higher than those of singletons after 2006. RR was around 1 and statistically not significant throughout all 5 years (RR = 0.90, 95% CI 0.78–1.05 or RR = 0.94, 95% CI 0.81–1.10 in total). 

The crude percentage of birth defects per total ART deliveries (including singleton stillbirth deliveries, singleton live-birth deliveries, stillbirth deliveries of multiples, and live-birth deliveries of multiples) were 0.64% (100/15,524) in 2004, 0.81% (133/16,385) in 2005, 1.13% (196/17,280) in 2006, 1.51% (268/17,761) in 2007, and 1.56% (320/20,542) in 2008. Throughout the 5 years in total, 1.16% of all neonates born after ART had birth defects (1,017/87,492, with 86,914 live deliveries, 578 stillbirth deliveries).

Although it became clear that there were a very high number of unknown/missing values of early neonatal death, the early neonatal mortality rate between singletons and multiples was compared for reference. The crude percentages of early neonatal death with birth defects are shown in Table 4. Although the percentages in multiples were consistently higher than in singletons, RR varied widely by year and was not necessarily significant. However, RR was statistically significant throughout the five years (RR = 2.68, 95% CI 1.52–4.70 or RR = 2.80, 95% CI 1.60–4.92 in total).

The crude early neonatal mortality with birth defects in ART and the general population (vital statistics) is shown in Table 5. Early neonatal mortality was slightly higher in ART (5.09 per 10,000 live births) than in the general population (3.86 per 10,000 live births) with marginal significance (RR = 1.32, 95% CI 1.00–1.75 in total).

The results of the logistic regression analyses are as follows. Neonatal outcome of birth defects after ART (whether stillbirths or live births) was strongly influenced by gestational week (*P* < .0001) and moderately by plurality (*P* = .02). Stillbirths were more frequently seen in singletons than in multiples. Early neonatal mortality of birth defects after ART (whether early neonatal death or not) was strongly influenced by gestational week (*P* < .0001).

## 4. Discussion

The present study for the first time showed the nationwide prevalence of birth defects after ART according to plurality in Japan, although there are several limitations to the study mentioned below. 

The percentage of birth defects per pregnancies and live births increased steadily by year, from 2004 to 2008, for both singletons and multiples. Although the definitive reason for this is unclear, one possible reason is that the precision of diagnosis improved.

In Japan, the percentage of birth defects per births were reported to be 1.77–1.95% from 2004 to 2006 according to the report of the ICBDMS Japan Center (http://www.icbdsrj.jp/2004data.html, in Japanese, accessed August 2011). This value is slightly higher than that of ART in the present study. However, direct comparison is impossible, because the present rate shown in Table 3 was for live births. Even if the percentage of birth defects per ART births were calculated assuming that stillbirths rate (22 or more weeks) according to plurality in ART and general population is equal and estimate the number of stillbirths using this rate according to plurality, the results were not dramatically changed (1.05-1.06% in singletons and 0.91–0.94% in multiples, calculation process not shown). Moreover, the method of data collection and diagnosis may be different. Fujii et al. [26] reported in the recent study that the percentage of birth defects rate in singleton births are 2.3% in IVF and 2.0% in a spontaneous singletons control, using the 2006 JSOG database. According to Fujii et al. [26], this data is not nationwide, which represents a large proportion of referral hospital data, with a large proportion of high-risk pregnancy population.

The present percentage of birth defects after ART are overall lower compared with other studies seen in many reviews [4, 6, 11]. Nevertheless, the main objective of this study was to evaluate the birth defect rate in multiple births compared to singletons within Japan and not to compare the birth defect rates across countries. Therefore, the comparison of birth defects in multiple births and singletons may be biased only if there is differential reporting according to plurality, which is not likely to occur [16]. 

The percentage of birth defects are clearly higher in multiple pregnancies than in singleton pregnancies with RR around 2 (Table 2), whereas the percentage of birth defects after ART in live ART births are equal in the two groups (Table 3). But caution is demanded, because pregnancies were counted by the number of pregnant women not by the number of fetuses. These results should be examined while considering family size (number of children in one family). If a certain family has two children after ART, the risk for birth defects is nearly the same, from the family's point of view, whether they are two singletons or one pair of twins. There exist no direct data indicating the mean number of ART children per family in Japan. According to the vital statistics in Japan, the mean number of children under 6 years old in one family, not stratified by the method of conception, was ca. 1.3 between 2004 and 2008. If the number of children in one family is the same whether the children are born after ART or spontaneously, this suggests that the risk of having birth defects in at least one baby in one family after ART may become higher in families with multiples with at least two children than in families with singletons. Therefore, the risk of birth defects after ART in multiples should be considered in not only the standpoint of births (babies), but maternity or family, as far as social impact of ART is concerned.

The higher percentage of abortion and/or stillbirth in singletons were observed, as shown in Table 1. The results of the logistic regression analysis for neonatal outcome of birth defects (whether stillbirths or live births) coincided with this result, although the reasons were unclear.

According to Bonduelle et al. [27], major birth defects after ICSI were found in 3.07% (46/1,499) of the singleton children and in 3.73% (50/1,341) of the children from multiple pregnancies. Major birth defects were found after IVF in 3.15% (49/1,556) of the singleton children and in 4.50% (63/1,399) of the children from multiple pregnancies. The rates of birth defects in multiples were significantly higher than in singletons in ICSI as well as in IVF (*P* < .05). 

Pinborg et al. [28] compared twins and singletons after ART (IVF/ICSI) using a Danish national birth cohort and their record linkage. They reported that the rate of major birth defects was not statistically significant between twins and singletons, whereas the total malformation rate (major and minor) was higher in twins than in singletons (*P* = .001). Other items, such as the risk for stillbirths, preterm delivery, low birthweight, use of cesarean sections, and admittance to a neonatal intensive care unit, were all higher in twins than in singletons, and the researchers concluded that neonatal outcome in IVF/ICSI twins is considerably poorer than in singletons.

As is well known, the Japanese perinatal and neonatal healthcare system and other factors such as universal access to healthcare, established referral mechanisms, and compliance of patients with pregnancy followup are all factors that make Japan one of the most developed countries in the field of maternal and child health [26]. For example, vital statistics show that the infant mortality (within one year after birth) rate is one of the lowest in the world (2.4 per 1,000 live births in 2009). Of them, birth defects have been the most serious cause of early neonatal mortality, neonatal mortality (within 28 days after birth), and infant mortality. The early neonatal mortality rate from 2004 to 2008 attributed to birth defects was consistently around 40%. Given the consistent increase of live births after ART, the impact of ART-related deaths due to birth defects is a public health concern.

As shown in Table 1, followup of neonates after ART is insufficient, with almost 26% unknown/missing values for early neonatal mortality. The present values are the minimum numbers, since more early neonatal deaths with birth defects definitely occur in complete dataset without unknown/missing values. The early neonatal mortality rate was slightly higher in ART than in the general population (RR = 1.32, 95% CI 1.00–1.75), as shown in Table 5, even using the minimum numbers of neonatal deaths with birth defects. The possibility that the mortality rate of neonates with birth defects after ART is higher than that in the general population, or non-ART population, cannot be denied. Since the present neonatal mortality rate after ART is a minimum estimate, precise information concerning definite followups after ART is essential.

This study has the following limitations, most of which could be attributed to the dataset, namely, the fact that individual information was obtained only from the subjects with birth defects after ART, not the total ART pregnancies. The first and greatest limitation is that the author could not control for confounding factors that can affect ART and/or birth defects, such as maternal age, parity, smoking [6, 29], and socioeconomic status, since these data on the general ART populations were not available. However, many research studies to date do not necessarily control for confounding factors [5]. Second, direct comparison between the present ART data and birth defects data, and vital statistics (early neonatal mortality rate) may be impossible, because the diagnostic or inclusion criteria for birth defects are not necessarily the same across the dataset. Third, all methods of ART, that is, IVF, ICSI, and so on, were treated as ART. Regarding this point, a recent meta-analysis [30] and national study [27] reported that the ICSI procedure represents no significant additional risks of major birth defects in addition to the risks involved in standard IVF. 

The author treated total birth defects, not each disease, in the present study to estimate the total impact of ART on birth defects. It is possible that each disease is related to methods of conception (IVF or ICSI), plurality (singletons or multiples), or other factors. For example, some birth defects in the urogenital system of boys are reported to be higher in ICSI than in IVF [29, 31]. Patent ductus arteriosus is strongly associated with preterm birth and may be higher in twins than in singletons [28, 32]. Further analysis of each disease is essential.

The increased risk of adverse outcome in ART multiples/twins is not limited to adverse neonatal outcome. ART twins are more likely to develop cerebral palsy and be hospitalized than ART singletons [33, 34]. According to the questionnaire survey by Pinborg et al. [35], speech development and physical health up to 4 years of age were significantly worse in twins than in singletons, and twins were more likely to have special needs and require surgical interventions than singletons. Maternal risks increased in ART twin pregnancies, as the women were more likely to be on sick leave and hospitalized during pregnancy [36]. 

The mothers of iatrogenic multiple-birth children are clearly older than the mothers of singletons in Japan [37]. Their advanced age makes the physical, mental, and social burden of rearing two babies at once even greater. This situation could increase the risk of maternal problems such as postpartum depression, difficulties with child rearing, and, in the worst case, child abuse [37–40]. 

These adverse outcomes of twin pregnancy after ART promoted an elective single embryo transfer policy for ART [41, 42]. While ART multiples decreased dramatically after 2006 in Japan [37], long-term social as well as medical followup for mothers of multiples and the multiples themselves was rarely performed. A standardized methodology of followup studies after ART should be established [43]. Considering the medical and social impacts of multiple births [37–40], there is an urgent need for a hospital-based monitoring system for fertility treatments, including not only ART but also non-ART treatment, and multiple births in Japan [25, 37]. Moreover, effective record linkage between data on fertility treatment, birth defects, and vital statistics is essential.

The risk of birth defects in ART live births is not significantly different between multiples and singletons, but the impact of birth defects after ART would be larger in families with multiples, since the mean number of children would be larger in these families compared to families with singletons. Proper followup for all families, especially for families with multiple pregnancies/births, after ART is needed.

## Figures and Tables

**Table 1:** Demographic and perinatal outcome data of the ART pregnancies with birth defects of known plurality.

		Singletons (*n* = 934)		Multiples (*n* = 229)		*P*
		*n *	%	*n *	%	
Birth year						
2004		88	71.5^a ^	35	28.5 ^a^	<0.001
2005		119	77.3	35	22.7	
2006		165	76.0	52	24.0	
2007		240	79.5	62	20.5	
2008		322	87.7	45	12.3	
Maternal age						
20–24		5	0.5	0	0.0	<0.001
25–29		85	9.1	30	13.1	
30–34		328	35.1	105	45.9	
35–39		394	42.2	82	35.8	
≥40		122	13.1	12	5.2	
Range			23–46		25–42	
Mean±SD			35.0 ± 4.1		33.5 ± 3.7	
Median			35.0		33.0	
Gestational weeks						<0.001
<12		4	0.4	0	0.0	
12–21		62	6.6	13	5.7	
22–27		20	2.1	13	5.7	
28–36		122	13.1	119	52.0	
37–41		613	65.6	78	34.1	
≥42		8	0.9	0	0.0	
Unknown/missing values		105	11.2	6	2.6	
Range			10–42		12–41	
Mean ± SD			36.1 ± 6.3		34.0±5.2	
Median			38.0		36.0	
Sex						
Male		453	48.5	111	48.5	n.s.
Female		350	37.5	91	39.7	
Unknown/missing values		131	14.0	27	11.8	
Neonatal outcome						
Abortion (<22 weeks)		133	14.2	14	6.1	<0.001
Stillbirths (22 ≤ weeks)		37	4.0	5	2.2	
Live-births		764	81.8	210	91.7	
Early neonatal death (neonatal death up to day 6 after birth)						
Yes		27	3.5	22	10.5	<0.001
No		539	70.6	153	72.9	
Unknown/missing values		198	25.9	35	16.7	

^a^ Percentage of singletons and multiples within each year were calculated.

n.s.: not significant.

Unknown/missing values were excluded in the statistic tests.

**Table 2:** Crude percentage of birth defects in ART pregnancy according to plurality.

Year	Singletons	Multiples	RR (95% CI)	Total
2004	0.43 (88/20,304)	0.97 (35/3,612)	2.24 (1.51–3.30)	0.50 (123/23,916)
2005	0.53 (119/22,524)	0.92 (35/3,784)	1.75 (1.20–2.55)	0.59 (154/26,308)
2006	0.67 (165/24,787)	1.52 (52/3,424)	2.28 (1.67–3.11)	0.77 (218/28,211)
2007	0.93 (240/25,944)	1.92 (62/3,221)	2.08 (1.58–2.74)	1.04 (304/29,165)
2008	1.06 (322/30,372)	2.10 (45/2,139)	1.98 (1.46–2.70)	1.13 (368/32,511)

Total	0.75 (934/123,931)	1.42 (229/16,180)	1.88 (1.60–2.13)	0.83 (1,167/140,111)^a^

Numbers of ART pregnancies according to plurality were presented in the JSOG annual report.

^a^Total number includes four subjects with unknown plurality.

**Table 3:** Crude percentage of birth defects in ART live-born babies according to plurality.

Year	Singletons	Multiples	RR (95% CI)	Total
2004	0.54 (69/12,851)*^a^*	0.51 (27/5,317)*^a^*	0.95 (0.61–1.47)*^a^*	0.52 (96/18,168)
	0.55 (69/12,562^)b^	0.48 (27/5,606)^b^	0.88 (0.56–1.37)^b^	
2005	0.67 (91/13,601)*^a^*	0.60 (33/5,511)*^a^*	0.90 (0.60–1.33)*^a^*	0.65 (124/19,112)
	0.68 (91/13,324)^b^	0.57 (33/5,788)^b^	0.83 (0.56–1.24)^b^	
2006	0.94 (140/14,876)^a^	1.04 (49/4,711)*^a^*	1.11 (0.80–1.53)*^a^*	0.96 (190/19,587)
	0.96 (140/14,649)^b^	0.99 (49/4,938)^b^	1.04 (0.75–1.44)^b^	
2007	1.28 (200/15,681)	1.43 (56/3,914)	1.12 (0.84–1.51)	1.32 (258/19,595)
2008	1.39 (264/19,034)	1.69 (45/2,670)	1.22 (0.89–1.66)	1.43 (310/21,704)

Total	1.00 (764/76,043)*^a^*	0.95 (210/22,123)*^a^*	0.94 (0.81–1.10)*^a^*	0.99 (978/98,166)*^c^*
	1.02 (764/75,250)^b^	0.92 (210/22,916)^b^	0.90 (0.78–1.05)^b^	

Numbers of ART live births according to plurality from 2004 to 2006 were estimated by the present author using JSOG annual report.

Numbers of ART live births according to plurality from 2007 to 2008 were presented in the JSOG annual report.

^a^Minimum estimation of numbers of multiples.

^b^Maximum estimation of numbers of multiples.

*^c^*Total number includes four subjects with unknown plurality.

**Table 4:** Crude early neonatal mortality rate of birth defects (per 1,000 ART live births).

Year	Singletons	Multiples	RR (95% CI)	Total
2004	0.16 (2/12,851)^a^	0.56 (3/5,317)^a^	3.63 (0.61–21.69)^a^	0.28 (5/18,168)
	0.16 (2/12,562)^b^	0.54 (3/5,606)^b^	3.36 (0.56–20.11)^b^	
2005	0.37 (5/13,601)^a^	0.54 (3/5,511)^a^	1.48 (0.35–6.19)^a^	0.42 (8/19,112)
	0.38 (5/13,324)^b^	0.52 (3/5,788)^b^	1.38 (0.33–5.78)^b^	
2006	0.47 (7/14,876)^a^	1.91 (9/4,711)^a^	4.06 (1.51–10.90)^a^	0.82 (16/19,587)
	0.48 (7/14,649)^b^	1.82 (9/4,938)^b^	3.81 (1.42–10.24)^b^	
2007	0.32 (5/15,681)	0.51 (2/3,914)	1.60 (0.31–8.26)	0.41 (8/19,595)
2008	0.42 (8/19,034)	1.87 (5/2,670)	4.46 (1.46–13.61)	0.60 (13/21,704)

Total	0.36 (27/76,043)^a^	0.99 (22/22,123)^a^	2.80 (1.60–4.92)^a^	0.51 (50/98,166)*^c^*
	0.36 (27/75,250)^b^	0.96 (22/22,916)^b^	2.68 (1.52–4.70)^b^	

Numbers of ART live births according to plurality from 2004 to 2006 were estimated by the present author using JSOG annual report.

Numbers of ART live births according to plurality from 2007 to 2008 were presented in the JSOG annual report.

^a^Minimum estimation of numbers of multiples.

^b^Maximum estimation of numbers of multiples.

^c^Total number includes one subject with unknown plurality.

**Table 5:** Crude early neonatal mortality rate as to birth defects (per 10,000 live births).

Year	ART	Total births	RR (95% CI)
2004	2.75 (5/18,168)	4.27 (474/1,110,721)	0.64 (0.27–1.56)
2005	4.19 (8/19,112)	3.78 (402/1,062,530)	1.11 (0.55–2.23)
2006	8.17 (16/19,587)	3.86 (422/1,092,674)	2.11 (1.28–3.48)
2007	4.08 (8/19,595)	3.95 (431/1,089,818)	1.03 (0.51–2.08)
2008	5.99 (13/21,704)	3.41 (372/1,091,156)	1.76 (1.01–3.05)

Total	5.09 (50/98,166)	3.86 (2,101/5,446,899)	1.32 (1.00–1.75)

Numbers of total ART live births were presented in the JSOG annual report.

Crude neonatal mortality rate of total births were calculated using vital statistics.
